# An optimal dietary sodium chloride supplemental level of broiler chicks fed a corn-soybean meal diet from 1 to 21 days of age

**DOI:** 10.3389/fvets.2022.1077750

**Published:** 2022-12-06

**Authors:** Weiyun Zhang, Bingxin Wu, Wei Wu, Xiaoyan Cui, Ding Li, Feiyu Gao, Tingting Li, Ling Zhu, Yanqiang Geng, Liyang Zhang, Yun Hu, Xugang Luo

**Affiliations:** ^1^Poultry Mineral Nutrition Laboratory, College of Animal Science and Technology, Yangzhou University, Yangzhou, China; ^2^Mineral Nutrition Research Division, State Key Laboratory of Animal Nutrition, Institute of Animal Science, Chinese Academy of Agricultural Sciences (CAAS), Beijing, China

**Keywords:** sodium chloride, optimal level, blood gas, serum parameters, jejunal morphology, organ index, broiler

## Abstract

Sodium chloride (NaCl) is usually added to diets to meet the Na and Cl requirements of broilers in the Chinese poultry industry, but the optimal dietary NaCl supplemental level was not well-established. The present study was conducted to estimate the optimal dietary NaCl supplemental level of broilers fed a corn-soybean meal diet from 1 to 21 days of age. A total of 490, 1-day-old Arbor Acres male broilers were fed a NaCl-unsupplemented corn-soybean meal basal diet (control) and the basal diet supplemented with 0.10, 0.20, 0.30, 0.40, 0.50 or 0.60% NaCl for 21 days. Regression analysis was conducted to evaluate the optimal dietary NaCl level using the best fitted broken-line or asymptotic models. As dietary supplemental NaCl levels increased, average daily gain (ADG), average daily feed intake (ADFI), blood partial pressure of CO_2_, total CO_2_, base excess and anion gap, blood concentrations of HCO_3_, Na and Cl, serum Na concentration, jejunal villus height (VH) and tibia ash content increased linearly and quadratically (*P* < 0.05), while feed/gain ratio, relative weights of heart, liver and kidney, blood K concentration, serum concentrations of K, uric acid and glucose, and osmotic pressure decreased linearly and quadratically (*P* < 0.05). The estimates of optimal dietary NaCl levels were 0.20−0.22% based on the best fitted broken-line or asymptotic models (*P* < 0.0001) of ADG, ADFI and feed/gain ratio, and 0.08−0.24% based on the best fitted broken-line or asymptotic models (*P* < 0.0001) of blood gas indices, serum parameters, jejunal VH, tibia ash content and organ indices. These results suggested that the optimal dietary NaCl supplemental level would be 0.24% for broilers fed the corn-soybean meal diet from 1 to 21 days of age, which is lower than the current dietary NaCl supplemental level (0.30%) in the Chinese broiler production.

## Introduction

Sodium (Na) and chlorine (Cl) are essential minerals for poultry, and play a critical role in regulating water-electrolyte metabolism, acid-base balance and maintaining osmotic pressure (OSM) (1–3). Optimal dietary balance of Na and Cl could improve chicken health and growth performance (4).

The current NRC recommended that both dietary Na and Cl requirements for broilers from 1 to 21 days of age are 0.20% (5), which are primarily based on the growth performance (4). Actually, 0.30% sodium chloride (NaCl) is usually added to diets to meet the Na and Cl requirements of broilers in the Chinese poultry industry. Nott and Combs (6) reported that the optimal NaCl levels in diets for broilers from 1 to 28 days of age were 0.25−0.30% based on the maximum growth. However, the growth performance parameters are usually not very sensitive in reflecting dietary Na and Cl requirements for modern fast-growing broilers. Therefore, it is necessary to reevaluate the optimal dietary NaCl supplemental level of broilers fed a conventional corn-soybean meal diet using more sensitive indices.

As important components of electrolytes in animals, Na, K, and Cl concentrations in blood can reflect the changes of electrolyte balance, and their concentrations within the normal range are important in maintaining physiological function (7–9). Oviedo-Rondón et al. (10) reported that the optimal dietary Cl supplemental level of broilers was 0.30% based on blood gas indices. Jiang et al. (11) reported that serum Na and OSM increased quadratically with increasing dietary supplemental Na and Cl levels. The Na and Cl participate in regulation of bone growth and mineralization (12). Murakami et al. (8) reported that the tibia ash content was a sensitive index for evaluating Na and Cl requirements of chicks fed a common corn-soybean basal diet, and the optimal Na and Cl requirements of broiler chicks from 1 to 21 days of age were 0.25 and 0.20%, respectively, based on the tibia ash content. Furthermore, long-term high-salt or low-salt diet could cause metabolic abnormalities and affect the growth and development of major organs in broilers (13, 14). In addition, the high-salt diet could increase the osmotic pressure of the intestinal contents and seriously affect the jejunal villus growth (15). However, it is unknown whether the above-mentioned blood gas indices, serum parameters, jejunal morphology and organ indices could be used to estimate the optimal dietary NaCl supplemental level of chicks fed the conventional corn-soybean meal diet.

Therefore, we hypothesized that blood gas indices, serum parameters, jejunal morphology and organ indices might be new and sensitive indices for evaluating the optimal dietary NaCl supplemental level of broiler chicks fed the conventional corn-soybean meal diet, and the optimal dietary NaCl supplemental level for broiler chicks estimated based on the above sensitive indices might be different from the current dietary NaCl supplemental level (0.30%) in the Chinese broiler production. The objective of the present study was to investigate the effect of dietary NaCl supplemental levels on growth performance, blood gas indices, serum parameters, jejunal morphology, tibia ash content and organ indices, and thus to select sensitive indices to evaluate the optimal dietary NaCl supplemental level for broilers fed the conventional corn-soybean meal diet from 1 to 21 days of age.

## Materials and methods

Animal experiments were approved by the Yangzhou University Animal Experiments Ethics Committee, with the permit number SYXK (Su) 2021-0027.

### Birds, diets, and experimental design

A total of 490, 1-day-old Arbor Acres (AA) commercial male broiler chicks were purchased from Jinghai Poultry Industry Group in Nantong, Jiangsu, China. The broilers were randomly divided into 1 of 7 groups with 7 replicate cages of 10 chicks each. All broilers were vaccinated with Newcastle disease vaccine and bronchitis combined vaccine at 7 days of age, and with infectious bursal disease virus vaccine at 14 days of age, respectively. All broilers were kept in a 24-h constant light and thermostatically controlled room equipped with stainless steel cages, feeders and plastic waterers (16). Dead birds, body weight, and feed intake were recorded to calculate average daily gain (ADG), average daily feed intake (ADFI), feed/gain ratio, and mortality from 1 to 21 days of age.

The basal corn-soybean meal diet (Table 1, containing Na: 0.02%, Cl: 0.08%) was formulated to meet or exceed the nutrient requirements (5) of starter broilers for all nutrients except for Na and Cl. Dietary treatments included the NaCl-unsupplemented corn-soybean meal basal diet (control) and the basal diet supplemented with 0.10, 0.20, 0.30, 0.40, 0.50 or 0.60% NaCl. The analyzed Na concentrations in diets are listed in Table 2.

**Table 1:** Composition and nutrient levels of the basal diet from 1 to 21 days of age (as-fed basis).

**Items**	**Content (%)**
**Ingredients**
Corn	52.43
Soybean meal	37.10
Soybean oil	5.10
${\text{CaHPO}}_{4}^{\text{a}}$	1.85
Limestone^a^	1.26
*DL*-methionine^a^	0.32
*L*-lysine·HCl^b^	0.05
Premix^c^	0.29
Zeolite powder + NaCl^d^	1.60
Total	100.00
**Nutrient composition**
Metabolizable energy, kcal/kg	3,001
Crude protein^e^	21.25
Lysine	1.15
Methionine	0.61
Methionine + cysteine	0.91
Ca^e^	0.99
Non-phytate P	0.45
Na^e^	0.02
K^e^	1.10

a Feed grade.

b Food grade.

c Provided per kilogram of diet: vitamin A (as all-trans retinol acetate), 12,000 IU; vitamin D_3_ (cholecalciferol), 4,500 IU; vitamin E (as all-rac-α-tocopherol acetate), 33 IU; vitamin K (as menadione sodium bisulfate), 3.0 mg; thiamin (as thiamin mononitrate), 3.0 mg; riboflavin, 9.6 mg; vitamin B_6_, 4.5 mg; vitamin B_12_, 0.03 mg; pantothenic acid calcium, 15 mg; niacin, 54 mg; folic acid, 1.5 mg; biotin, 0.15 mg; choline (as choline chloride), 700 mg; Cu (CuSO_4_·5H_2_O), 8 mg; Zn (ZnSO_4_·H_2_O), 60 mg; Mn (MnSO_4_·H_2_O), 110 mg; Fe (FeSO_4_·H_2_O), 40 mg; I [Ca(IO_3_)_2_·H_2_O], 0.35 mg; Se (Na_2_SeO_3_), 0.15 mg.

d NaCl supplements added in place of equivalent weights of zeolite powder.

e Values determined by analysis. Each value based on triplicate determinations.

**Table 2:** Analyzed Na concentrations in diets for broilers from 1 to 21 days of age^a^.

**Supplemental NaCl (%)**	**Analyzed Na contents (%)**
0.00	0.02
0.10	0.06
0.20	0.10
0.30	0.14
0.40	0.18
0.50	0.22
0.60	0.26

a Values of analyzed Na contents are based on triplicate determinations.

### Sample collections and preparations

The feed ingredient and diet samples were collected for analyzes of Ca, K, Na or dietary crude protein (CP) concentrations. The tap water was collected for analyzes of Ca, Na, and K concentrations (contents of these minerals in tap water were analyzed to be <44 μg/ml).

The initial weight of water was recorded and the remaining water was measured at 8:00 o'clock the next morning daily throughout the entire experimental period, and the differences between the water weight determined at the beginning and end of each measurement were used to calculate the water consumption (ml/day). Five days before the end of the experiment, all excrements in each replicate cage were collected and weighed, dried at 60°C until constant weight to determine the moisture content.

At 21 days of age, three birds from each replicate cage were chosen according to average body weight of each cage. Whole blood samples were taken into lithium heparin tubes from each of two birds *via* the wing veins for blood gas analysis. Another blood samples were collected from each bird into tubes without the anticoagulant, and centrifuged to harvest serum for analyzes of Na, Cl, K, glucose (GLU), and uric acid concentrations. Serum samples from three chicks in each cage were pooled into one sample in equal ratios before analyzes. The selected birds in each cage were killed by cervical dislocation, and heart, liver, spleen, lung and kidney samples were weighed and divided by living body weights to calculate organ indices. The right tibia was peeled and frozen at −20°C for analysis of the tibia ash content. The jejunal sample (2 cm in length) was collected from the midpoint of the jejunum of a chick and fixed in 4% formaldehyde for the subsequent morphological examination (17).

### Measurements of Na, K, Ca, and CP concentrations

The concentrations of Na, K, and Ca in tap water, feed ingredients and diets, CP in feed ingredients or diets were determined as described previously (18, 19).

### Determinations of blood gas indices and serum parameters

The whole blood samples in the lithium heparin tubes were measured with a blood gas analyzer (VS4-6163, VetStat, USA) for pH, total CO_2_ (TCO_2_), partial pressure of CO_2_ (PCO_2_), anion gap (AG), base excess (BE), and the concentrations of H, HCO_3_, Na, Cl, and K. An automatic biochemical analyzer (7180, Hitachi, Japan) was used to measure serum K, Na, Cl, glucose (GLU), and uric acid concentrations, and then calculated the serum OSM (20).

### Measurements of tibia ash content and jejunal morphological indices

The tibia ash content was determined as described previously (21). The jejunal morphometric parameters were photographed at ×40 magnification under the computer-aided light microscope (CKX53, OLYMPUS, Japan). The total villi height (VH) and crypt depth (CD) were measured using ImageJ software, and the villi-crypt ratio (VH/CD) was calculated according to the procedure of Zhang et al. (22). The values of means from 10 to 20 adjacent and vertically orientated villi and crypts were used for further analyzes.

### Statistical analyzes

The data in this study were submitted to one-way ANOVA using the general linear model of SAS 9.4 (SAS Institute Inc., Cary, NC) (23). Differences among means were tested with the LSD method. Data on the percentage of mortality of broilers were converted to arcsine for analysis. Each replicate cage was an experimental unit. The linear and quadratic responses of responsive indices to added NaCl levels were tested using orthogonal comparisons. Regression analyzes of broken-line, quadratic and asymptotic models were performed, and the best fitted models between responsive criteria and added NaCl levels were used to estimate the optimal added NaCl levels (the break point from the broken-line model or 95% of the maximum response from the asymptotic model) for broiler chicks (24). The *P* < 0.05 was considered to be statistically significant.

## Results

### Growth performance, mortality, average daily water intake, and excreta moisture content

The ADG, ADFI, average daily water intake and excreta moisture content increased linearly and quadratically (*P* < 0.0001), while the feed/gain ratio decreased linearly and quadratically (*P* < 0.0001) as dietary supplemental NaCl levels increased (Table 3). The ADG and ADFI reached a plateau at the supplemental NaCl level of 0.30%, and the feed/gain ratio reached a plateau at the supplemental NaCl level of 0.20%. The birds fed on the basal diet without the NaCl supplementation showed the highest mortality rate.

**Table 3:** Effect of dietary NaCl supplemental level on growth performance, mortality, average daily water intake and excreta moisture content of broilers from 1 to 21 days of age^1^.

**Supplemental NaCl** ** (%)**	**ADG** ** (g/day)**	**ADFI** ** (g/day)**	**Feed/gain ratio** ** (g/g)**	**Mortality** ** (%)**	**Average daily water intake** ** (ml/days)**	**Excreta moisture content** ** (%)**
0.00	2.93^d^	12.4^d^	4.08^a^	48.57^a^	18.2^e^	39.1^b^
0.10	15.26^c^	28.0^c^	1.81^b^	1.43^b^	69.4^d^	74.9^a^
0.20	31.43^b^	44.7^b^	1.44^b^	1.43^b^	112.3^c^	75.5^a^
0.30	33.95^a^	46.8^ab^	1.38^b^	2.86^b^	115.0^bc^	74.6^a^
0.40	34.12^a^	47.9^a^	1.38^b^	0.00^b^	114.4^bc^	74.4^a^
0.50	34.18^a^	47.4^a^	1.39^b^	1.43^b^	118.6^ab^	75.8^a^
0.60	34.08^a^	48.0^a^	1.41^b^	2.86^b^	123.2^a^	77.4^a^
SEM	0.49	0.9	0.15	3.31	1.8	1.0
***P*** **-value**						
NaCl	<0.0001	<0.0001	<0.0001	<0.0001	<0.0001	<0.0001
Linear	<0.0001	<0.0001	<0.0001	0.0756	<0.0001	<0.0001
Quadratic	<0.0001	<0.0001	<0.0001	0.6121	<0.0001	<0.0001

ADG, average daily gain; ADFI, average daily feed intake.

^a−*e*^Means with different superscripts within a column differ (P < 0.05).

^1^Data represent the means of 6/7 replicate cages (n = 6/7).

### Blood gas indices

The PCO_2_, TCO_2_, BE and AG, and the concentrations of HCO_3_, Na, and Cl in blood increased linearly and quadratically (*P* < 0.0004), while the K concentration in blood decreased linearly and quadratically (*P* < 0.0001) with the increase of dietary NaCl levels (Table 4). The PCO_2_, TCO_2_, BE and the concentrations of HCO_3_, K, Na, and Cl in blood reached a plateau at supplemental NaCl levels of about 0.20−0.60%.

**Table 4:** Effect of dietary NaCl supplemental level on blood gas indices of broilers on day 21^1^.

**Supplemental NaCl** ** (%)**	**pH**	**PCO_2_ (mmHg)**	**HCO_3_ (mmol/L)**	**TCO_2_ (mmol/L)**	**BE (mmol/L)**	**Na (mmol/L)**	**Cl (mmol/L)**	**H (nmol/L)**	**AG (mmol/L)**	**K (mmol/L)**
0.00	7.39	29.0^c^	16.3^c^	17.1^c^	−4.68^b^	121^c^	106^c^	41.0	9.83^b^	9.98^a^
0.10	7.39	33.2^b^	18.0^b^	18.9^b^	−3.26^b^	131^b^	109^b^	41.1	13.18^a^	8.48^b^
0.20	7.41	37.4^a^	21.1^a^	22.1^a^	−0.73^a^	145^a^	115^a^	39.3	13.84^a^	5.01^c^
0.30	7.42	36.8^a^	21.2^a^	22.2^a^	−0.39^a^	146^a^	116^a^	38.9	13.64^a^	4.70^c^
0.40	7.4	37.7^a^	21.0^a^	22.0^a^	−0.99^a^	146^a^	116^a^	39.9	13.94^a^	4.79^c^
0.50	7.39	39.2^a^	21.9^a^	22.9^a^	−0.39^a^	145^a^	115^a^	39.8	13.15^a^	4.81^c^
0.60	7.39	38.3^a^	21.5^a^	22.6^a^	−0.39^a^	146^a^	115^a^	40.4	13.58^a^	4.82^c^
SEM	0.01	1.0	0.4	0.5	0.50	1	1	1.1	0.48	0.22
***P*** **-value**										
NaCl	0.6207	<0.0001	<0.0001	<0.0001	<0.0001	<0.0001	<0.0001	0.7800	<0.0001	<0.0001
Linear		<0.0001	<0.0001	<0.0001	<0.0001	<0.0001	<0.0001		<0.0001	<0.0001
Quadratic		0.0003	<0.0001	<0.0001	0.0003	<0.0001	<0.0001		<0.0001	<0.0001

PCO_2_, partial pressure of CO_2_; TCO_2_, total CO_2_; BE, base excess; AG, anion gap.

^a−*c*^Means with different superscripts within a column differ (P < 0.05).

^1^Data represent the means of 6/7 replicate cages (n = 6/7).

### Serum parameters and osmotic pressure

As the levels of supplemental NaCl increased, the OSM and the concentrations of K, uric acid and GLU in serum decreased linearly and quadratically (Table 5, *P* < 0.0001), while the Na concentration in serum increased linearly and quadratically (*P* < 0.0004). The uric acid concentration in serum reached a plateau at supplemental NaCl levels of about 0.20−0.60%; the GLU concentration in serum reached a plateau at supplemental NaCl levels of about 0.20–0.50%; and the K concentration and OSM in serum reached the lowest point at the supplemental NaCl level of 0.20%.

**Table 5:** Effect of dietary NaCl supplemental level on serum biochemical variables of broilers on day 21^1^.

**Supplemental NaCl** ** (%)**	**Na** ** (mmol/L)**	**Cl** ** (mmol/L)**	**K** ** mmol/L**	**GLU** ** (mmol/L)**	**Uric acid** ** (mmol/L)**	**OSM** ** (mmol/L)**
0.00	146^b^	115^a^	6.75^a^	13.85^a^	1.45^a^	321^a^
0.10	146^b^	115^ab^	6.34^b^	11.39^b^	0.53^b^	317^b^
0.20	146^b^	114^abc^	4.44^d^	6.99^c^	0.24^c^	307^d^
0.30	146^b^	114^abc^	4.61^cd^	6.57^c^	0.23^c^	308^d^
0.40	146^a^	113^cd^	4.74^c^	5.93^c^	0.24^c^	308^cd^
0.50	146^b^	113^d^	4.76^c^	6.40^c^	0.21^c^	308^cd^
0.60	148^a^	114^bcd^	4.69^cd^	5.79^c^	0.22^c^	311^c^
SEM	0.2	0.4	0.09	0.57	0.04	1
***P*** **-value**						
NaCl	0.0001	0.0010	<0.0001	<0.0001	<0.0001	0.0010
Linear	0.0003	<0.0001	<0.0001	<0.0001	<0.0001	<0.0001
Quadratic	0.0003	0.1778	<0.0001	<0.0001	<0.0001	<0.0001

GLU, glucose content in serum; OSM, osmotic pressure in serum.

^a−*d*^Means with different superscripts within a column differ (P < 0.05).

^1^Data represent the means of 6/7 replicate cages (n = 6/7).

### Jejunal morphological characteristics, tibia ash content, and organ indices

As the levels of supplemental NaCl increased, the jejunal CD and tibia ash content increased linearly and quadratically (Table 6, *P* < 0.002), but the jejunal VH only increased quadratically (Table 6, *P* < 0.0001). In addition, the liver and kidney indices decreased linearly and quadratically (Table 6, *P* < 0.0001), while the heart index only decreased quadratically (Table 6, *P* < 0.0001) as supplemental NaCl levels increased. The jejunal CD, tibia ash content and heart index reached a plateau at supplemental NaCl levels of about 0.20−0.50%. Liver and kidney indices reached the lowest point at the supplemental NaCl level of 0.20%, and the jejunal VH reached the highest point at the supplemental NaCl level of 0.20%.

**Table 6:** Effect of dietary NaCl supplemental level on jejunal VH, CD, VH/CD, tibia ash content and organ indices of broilers on day 21^1^.

**Supplemental NaCl** ** (%)**	**Jejunal VH** ** (μm)**	**Jejunal CD** ** (μm)**	**VH/CD ratio** ** (μm/μm)**	**Tibia ash** ** (%)**	**Heart index** ** (g/kg)**	**Liver index** ** (g/kg)**	**Spleen index** ** (g/kg)**	**Lung index** ** (g/kg)**	**Kidney index** ** (g/kg)**
0.00	695^d^	117^c^	5.71	41.6^d^	8.04^a^	31.8^a^	0.86	8.37	17.85^a^
0.10	871^cd^	154^bc^	5.92	48.0^c^	5.32^d^	21.8^bc^	0.96	6.64	9.10^b^
0.20	1187^a^	191^ab^	4.20	49.4^b^	6.23^bc^	20.7^c^	0.93	8.07	6.94^c^
0.30	1102^ab^	202^a^	5.55	50.2^ab^	6.28^bc^	21.6^bc^	0.95	7.47	7.49^c^
0.40	1044^abc^	216^a^	5.02	50.7^ab^	6.19^c^	21.2^c^	0.92	7.72	7.42^c^
0.50	903^bcd^	199^ab^	4.95	50.5^ab^	6.70^bc^	22.3^bc^	0.87	7.38	7.65^c^
0.60	908^bcd^	193^ab^	4.35	51.0^a^	7.01^b^	23.5^b^	1.08	7.18	7.21^c^
SEM	66	14	0.47	0.4	0.26	0.7	0.05	0.39	0.36
***P*** **-value**									
NaCl	0.0007	0.0002	0.1112	<0.0001	<0.0001	<0.0001	0.1076	0.0605	<0.0001
Linear	0.1400	<0.0001		<0.0001	0.7860	<0.0001			<0.0001
Quadratic	<0.0001	0.0012		<0.0001	<0.0001	<0.0001			<0.0001

VH, villus height; CD, crypt depth.

^a−*d*^Means with different superscripts within a column differ (P < 0.05).

^1^Data represent the means of 4–7 replicate cages (n = 4–7).

### Optimal dietary supplemental NaCl levels

The optimal dietary supplemental NaCl levels of broilers from 1 to 21 days of age as estimated by the non-linear regression analyzes were shown in Table 7. Based on the best fitted broken-line or asymptotic models (*P* < 0.0001) of the ADG, ADFI and feed/gain ratio, the optimal dietary supplemental NaCl levels were 0.20−0.22%; based on the best fitted broken-line or asymptotic models (*P* < 0.0001) of the blood gas parameters and serum parameters, the optimal dietary supplemental NaCl levels were 0.08−0.24%; based on the best fitted broken-line or asymptotic models (*P* < 0.0001) of the jejunal VH and tibia ash content, the optimal dietary supplemental NaCl level were 0.21 and 0.11%, respectively; and based on the best fitted broken-line models (*P* < 0.0001) of the organ indices, the optimal dietary supplemental NaCl levels were 0.09−0.12% for broilers fed the corn-soybean meal diet from 1 to 21 days of age.

**Table 7:** The optimal dietary NaCl levels of chicks during 1–21 days of age as estimated based on the best fitted broken-line or asymptotic models.

**Dependent variable**	**Regression equation^a^**	**Coefficient of determination (*R*^2^)**	***P*-value**	**Optimal added NaCl level (%)**
ADG	Y = 2.20 + 143.00 X (0.00 ≤ X ≤ 0.22)	0.9858	<0.0001	0.22
	Y = 2.10 + 143.45 X (0.22 <X ≤ 0.60)			
ADFI	Y = 12.17 + 161.80 X (0.00 ≤ X ≤ 0.21)	0.9721	<0.0001	0.21
	Y = 11.54 – 164.74 X (0.21 <X ≤ 0.60)			
Feed/gain ratio	Y = 1.39 + 2.70 × e ^(−18.15*X*)^	0.8688	<0.0001	0.20
PCO_2_ in blood	Y = 38.81 – 9.91 × e ^(−6.76*X*)^	0.6504	<0.0001	0.24
HCO_3_ in blood	Y = 16.08 + 23.82 X (0.00 ≤ X ≤ 0.20)	0.7489	<0.0001	0.20
	Y = 15.70 + 25.69 X (0.20 <X ≤ 0.60)			
TCO_2_ in blood	Y = 16.84 + 25.21 X (0.00 ≤ X ≤ 0.20)	0.7588	<0.0001	0.20
	Y = 16.42 + 27.29 X (0.20 <X ≤ 0.60)			
Cl in blood	Y = 105.00 + 49.31 X (0.00 ≤ X ≤ 0.23)	0.8440	<0.0001	0.23
	Y = 105.59 + 46.73 X (0.23 <X ≤ 0.60)			
Na in blood	Y = 120.80 + 117.10 X (0.00 ≤ X ≤ 0.22)	0.9257	<0.0001	0.22
	Y = 121.30 + 114.81 X (0.22 <X ≤ 0.60)			
K in blood	Y = 10.35 – 25.11 X (0.00 ≤ X ≤ 0.23)	0.9087	<0.0001	0.23
	Y = 10.27 – 24.72 X (0.23 <X ≤ 0.60)			
BE in blood	Y = −4.86 + 19.75 X (0.00 ≤ X ≤ 0.21)	0.616	<0.0001	0.21
	Y = −4.99 + 20.33 X (0.21 <X ≤ 0.60)			
AG in blood	Y = 13.62 – 3.80 × e ^(−22.58*X*)^	0.5466	<0.0001	0.08
Uric acid in serum	Y = 1.45 – 9.17 X (0.00 ≤ X ≤ 0.13)	0.9355	<0.0001	0.13
	Y = 1.45 – 9.21 X (0.13 <X ≤ 0.60)			
GLU in serum	Y = 14.17 – 34.29 X (0.00 ≤ X ≤ 0.22)	0.8066	<0.0001	0.22
	Y = 14.59 – 36.16 X (0.22 <X ≤ 0.60)			
K in serum	Y = 7.00 – 11.54 X (0.00 ≤ X ≤ 0.21)	0.8762	<0.0001	0.21
	Y = 6.94 – 11.25 X (0.21 <X ≤ 0.60)			
OSM in serum	Y = 322.10 – 68.99 X (0.00 ≤ X ≤ 0.22)	0.8206	<0.0001	0.22
	Y = 320.17 – 60.27 X (0.22 <X ≤ 0.60)			
Jejunal VH	Y = 676 + 2389 X (0.00 ≤ X ≤ 0.21)	0.4481	<0.0001	0.21
	Y = 848 + 1665 X (0.21 <X ≤ 0.60)			
Tibia ash	Y = 50.68 – 9.07 × e ^(−11.57*X*)^	0.9000	<0.0001	0.11
Heart index	Y = 8.04 – 28.89 X (0.00 ≤ X ≤ 0.09)	0.5680	<0.0001	0.09
	Y = 7.80 – 26.10 X (0.09 <X ≤ 0.60)			
Liver index	Y = 31.76 – 99.43 X (0.00 ≤ X ≤ 0.12)	0.8257	<0.0001	0.12
	Y = 31.02 – 93.11 X (0.12 <X ≤ 0.60)			
Kidney index	Y = 17.85 – 87.46 X (0.00 ≤ X ≤ 0.12)	0.9425	<0.0001	0.12
	Y = 17.77 – 86.84 X (0.12 <X ≤ 0.60)			

ADG, average daily gain; ADFI, average daily feed intake; PCO_2_, partial pressure of CO_2_; TCO_2_, total CO_2_; BE, base excess; AG, anion gap; GLU, glucose; OSM, osmotic pressure; VH, villus height.

^a^Y, measurement of index; X, supplemental NaCl level (%) in the basal diet. Regression equations based on added NaCl level (%).

## Discussion

The key findings of the present study have demonstrated that the PCO_2_, TCO_2_, AG, BE, and the concentrations of HCO_3_, Na, Cl, and K in blood, the OSM and the concentrations of uric acid, GLU and K in serum, jejunal VH, heart, liver and kidney indices are new and sensitive criteria for estimating optimal dietary supplemental NaCl levels of broilers fed the corn-soybean meal diet from 1 to 21 days of age. Moreover, the optimal dietary supplemental NaCl levels were estimated to be 0.20−0.22% to support the best growth, 0.13−0.22% to support the serum ion balance, 0.08−0.24% to support the physiological balance in the blood, and 0.09−0.21% to support the jejunal villus, tibia and organ development of broilers fed the corn-soybean meal diet from 1 to 21 days of age based on the best fitted broken-line or asymptotic models. Therefore, it is suggested that the optimal dietary NaCl level to meet all needs of the modern and rapidly growing broilers for the above NaCl metabolisms would be 0.24%, which is lower than the current supplemental NaCl level (0.30%) in the Chinese broiler production. The above findings have supported our hypotheses and been not reported before, and also provided scientific bases for precisely adding the NaCl to the broiler diets in the Chinese broiler production.

The growth performance parameters were the early and commonly-used criteria to assess Na and Cl requirements in animals (4, 10). Previous studies demonstrated that broilers fed a diet deficient in Na and Cl decreased ADG and ADFI (2, 25). In the present study, we found that ADFI and ADG increased linearly and quadratically, while feed/gain ratio decreased linearly and quadratically with increasing dietary supplemental NaCl levels. And dietary supplemental 0.30−0.60% NaCl levels were enough to maintain normal growth for broiler chicks up to 21 days of age. Besides, in our current study, as the supplemental NaCl levels increased, the average daily water intake and excreta moisture content increased linearly and quadratically. As the water intake and the excreta moisture content could not accurately reflect the Na and Cl status of broilers, these two parameters were not suitable for evaluating the optimal dietary NaCl supplemental level of broiler chicks. In addition, broilers fed on the basal diet without the NaCl supplementation showed the highest mortality rate, which is in agreement with the previous reports (26–29), indicating that Na and Cl are essential minerals for poultry health and growth.

The Na and Cl jointly control the volume of extracellular fluid and osmotic pressure, and regulate the body fluid and acid-base balance (26, 30). The blood gas parameters are important indicators to evaluate the metabolic acid-base imbalance in the body (31, 32). A previous study showed that blood gas parameters could reflect the bioavailability of Na and Cl in broiler chicks (10). In the present study, the NaCl-unsupplemented diet (control) had an acidogenic effect on chicken metabolism, while supplemental NaCl levels significantly increased Na and Cl concentrations, and CO_2_ storage in blood, thus causing higher HCO_3_ concentration and BE in blood. Based on PCO_2_, TCO_2_, HCO_3_, BE, and AG parameters in blood, it was estimated that broilers fed diets containing 0.20−0.60% added NaCl could maintain acid-base balance in the body. In addition, Na and Cl are important components of blood crystalloid osmotic pressure, and the crystalloid osmotic pressure of blood could normally maintain the relative stable state of the internal environment. Salt intake could increase serum Na and uric acid concentrations, and then lead to changes in serum ionic parameters, glucose metabolism and OSM (33–36). Yu et al. (26) reported that the OSM and the concentrations of K, Na, uric acid, and GLU in the serum of broilers were affected linearly or quadratically with the increase of dietary supplemental Na and Cl levels. The present study also showed that the OSM and the concentrations of K, Na, uric acid and GLU in the serum were decreased linearly and quadratically as the levels of supplemental NaCl increased. Moreover, the above results show that the OSM and the concentrations of K, Na, uric acid and GLU in the serum are new and sensitive indices for estimating optimal dietary supplemental NaCl levels of broiler chicks, which has been not reported before. Based on the OSM and the serum K, uric acid and GLU concentrations, supplemental NaCl levels of 0.20% or more could support the OSM, serum ion and internal environment balance of broilers. Therefore, our findings along with those of others have clearly indicated that the blood gas parameters and serum biochemical indicators could be used as sensitive biomarkers for estimating optimal dietary supplemental NaCl levels of broilers. In the present study, although the serum Na concentration increased linearly and quadratically with the increase of dietary NaCl levels, the coefficient of determination *R*^2^ (0.3783) was relatively too low to evaluate the optimal dietary NaCl supplemental level of broiler chicks.

The Na and Cl balance is one part of the internal environment homeostasis that is necessary to maintain the health of the small intestine. Wang et al. (15) reported that the high-salt diet seriously affected the small intestinal villi growth in rats. In our present study, the broilers fed on the basal control diet without the NaCl addition had the lowest jejunal VH and CD. Besides, as the supplemental NaCl levels increased, the jejunal VH and CD increased linearly and quadratically. As the longer CD was unfavorable for the intestinal absorption of nutrients, it was not suitable for evaluating the optimal dietary NaCl supplemental level of broiler chicks. The Na and Cl participate in regulation of bone growth and mineralization (12). Taheri et al. (37) reported that the tibia ash content significantly increased with the increase of dietary NaCl levels. In our current study, tibia ash content increased linearly and quadratically as dietary supplemental NaCl levels increased, and reached a plateau at the supplemental NaCl level of 0.30%. The Na and Cl are mainly distributed in body fluids and soft tissues, and they are closely related to the regulation of water-salt balance in the body. Previous studies have suggested that Na intake could increase blood pressure in mice (38, 39). In addition, the long-term low salt diet increased the systolic blood pressure, cardiomyocyte size, interventricular septum thickness, renal edema and plasma angiotensin II levels, and chronically high levels of angiotensin II could lead to varying degrees of multiorgan damages (15, 40, 41). In our current study, the broilers fed with the NaCl-deficient diet had higher heart, liver and kidney indices, and they were decreased quadratically with increasing NaCl supplemental levels. Similar results were observed in the previous study of Jankowski et al. (42). These effects might be related to changes in blood pressure and angiotensin II caused by supplemental NaCl levels (40, 41), and the exact reasons need to be further investigated. Therefore, the above results demonstrated that jejunal VH and tibia ash content, heart, liver and kidney indices could be used as sensitive criteria to evaluate optimal dietary NaCl supplemental levels of broiler chicks from 1 to 21 days of age. And dietary supplemental 0.20−0.60% NaCl levels were sufficient for the normal development of tibia, jejunal villi, and organs.

The current study showed that the optimal dietary NaCl supplemental levels were 0.08−0.24% based on the best fitted broken-line or asymptotic models of new and sensitive indices of blood gas indices, serum parameters, jejunal morphology and organ indices of broilers. Although these results might not accurately reflect dietary Cl requirement of broiler chicks, the present study suggested that the optimal dietary NaCl supplemental level of 0.24% (a total 0.11% Na and 0.23% Cl with a Na/Cl ratio of 1:2.09 in the diet) could support all needs for the optimal growth, serum ion balance, blood acid-base balance, as well as jejunal villus and organ developmental needs of broilers fed a conventional corn-soybean meal diet from 1 to 21 days of age. The total 0.11% Na in the diet is less than the dietary Na requirement (0.20%), but the total 0.23% Cl is higher than the dietary Cl requirement (0.20%) as recommended by NRC (5). Therefore, further studies need be carried out to precisely reevaluate the optimal dietary Cl requirement of broilers under dietary Na/Cl ratio of 1:1 in order to minimize the excess of dietary Cl.

In conclusion, our data indicate that the PCO_2_, TCO_2_, AG, BE, and the concentrations of HCO_3_, Na, Cl, and K in blood, the OSM and the concentrations of uric acid, GLU and K in serum, jejunal VH, heart, liver and kidney indices can be used as new and sensitive criteria to estimate optimal dietary supplemental NaCl levels of broiler chicks. The optimal dietary NaCl supplemental level was suggested to be 0.24% for broilers fed the corn-soybean meal diet from 1 to 21 days of age, which is lower than the current dietary NaCl supplemental level (0.30%) in the Chinese broiler production.

## Data availability statement

The original contributions presented in the study are included in the article/supplementary material, further inquiries can be directed to the corresponding author/s.

## Ethics statement

All experimental procedures were approved by the Animal Care Committee of the Department of Animal Science and Technology of Yangzhou University, Yangzhou, China (permit number: SYXK (Su) 2021-0027) and conducted in accordance with the guidelines of the Animal Use Committee of the Chinese Ministry of Agriculture (Beijing, China).

## Author contributions

WZ: original draft preparation. DL: software. WW and BW: methodology. XC and FG: investigation. YG and LZhu: formal analysis. TL and LZha: validation and supervision. YH: review. XL: review and supervision. All authors contributed to the article and approved the submitted version.

## Funding

This study was funded by the Jiangsu Shuang Chuang Ren Cai program (JSSCRC2021541), the Jiangsu Shuang Chuang Tuan Dui program (JSSCTD202147), and the Initiation Funds of Yangzhou University for Distinguished Scientists.

## Conflict of interest

The authors declare that the research was conducted in the absence of any commercial or financial relationships that could be construed as a potential conflict of interest.

## Publisher's note

All claims expressed in this article are solely those of the authors and do not necessarily represent those of their affiliated organizations, or those of the publisher, the editors and the reviewers. Any product that may be evaluated in this article, or claim that may be made by its manufacturer, is not guaranteed or endorsed by the publisher.
